# Myeloperoxidase Anti-Neutrophil Cytoplasmic Antibody (MPO-ANCA)-Associated Vasculitis With Rare Clinical Manifestations After SARS-CoV-2 Multisystem Inflammatory Syndrome in a 14-Year-Old Boy

**DOI:** 10.7759/cureus.66859

**Published:** 2024-08-14

**Authors:** Vasiliki M Kymioni, Filippos Filippatos, Vasiliki Karava, Konstantinos Kakleas

**Affiliations:** 1 Paediatrics, Agia Sofia Children's Hospital, Athens, GRC; 2 Nephrology, Agia Sofia Children's Hospital, Athens, GRC; 3 Allergy and Immunology, Agia Sofia Children's Hospital, Athens, GRC

**Keywords:** clinical autoimmunity, mis-c in children, • anca- associated vasculitis, sars-cov-2, mpo-anca

## Abstract

Myeloperoxidase antineutrophil cytoplasmic antibody (MPO-ANCA) microscopic polyangiitis is a rare but life-threatening small vessel vasculitis in childhood that affects multiple systems. Emerging clinical evidence suggests a possible association between SARS-CoV-2 infection or multisystem inflammatory syndrome in children (MIS-C) as well as the futuredevelopment of autoimmune diseases. A 14-year-old boy with a diagnosis of MIS-C two years prior to presentation was admitted to our hospital due to edema and left lower limb joint pain along with concomitant upper surface petechia. The patient had a positive higher SARS-CoV-2 IgG than MIS-C diagnosis titers and MPO-ANCA-positive antibody titers. Kidney biopsy favored a pauci-immune crescentic glomerulonephritis. Restrictive lung disease with concomitant diffusion abnormalities was also observed. Pancreatitis and gastrointestinal wall edema were additional clinical manifestations. SARS-CoV-2 breakthrough infection and MIS-C could contribute to the onset of autoimmune vasculitis through various immunological mechanisms. Further research is still needed to elucidate the role of SARS-CoV-2 in the pathophysiology of newly diagnosed autoimmune vasculitis.

## Introduction

Myeloperoxidase anti-neutrophil cytoplasmic antibody (MPO-ANCA)-associated microscopic polyangiitis (MPA) is a rare but life-threatening small vessel vasculitis in childhood [1]. Clinical manifestations are variable and involve several systems such as the renal, respiratory, ear, nose, throat, and gastrointestinal systems [1]. The estimated incidence of MPA ranges from 9.0 to 94.0 cases per million individuals, with an average rate of 0.5 to 24.0 cases per million person-years [1]. White populations have a 200% higher occurrence of granulomatosis with polyangiitis (GPA), MPA, and eosinophilic granulomatosis with polyangiitis (EGPA), with an equal distribution between sexes [2-4].

There are four distinct MPO and proteinase 3 (PR3) double-positive ANCA angiitis types: idiopathic, drug-induced, autoimmune, and immune-disrupting types not associated with ANCA-associated vasculitis (AAV) [5]. Based on a study of over one hundred double-positive ANCA confirmed cases, there is a significant variation in terms of clinical and immunopathological manifestations; approximately half of the patients have developed AAV [5].

This report presents a case of a newly diagnosed MPO-ANCA angiitis in a patient with a previous medical history of multisystem inflammatory syndrome in children (MIS-C) and higher SARS-CoV-2 antibody titers compared to MIS-C diagnosis.

## Case presentation

A 14-year-old boy was admitted to the hospital due to a month-long history of fluctuating edema and joint pain of the left lower limb, along with concomitant upper surface petechia. The patient reported pain during active and passive movement of the joint which required anti-inflammatory medication for symptom relief. No constitutional symptoms, such as fever, malaise, anorexia, and weight loss, were reported. From the previous medical history, he presented with fever and pancreatitis and was diagnosed with MIS-C (SARS-CoV-2 antibody titers: 780 AU/ml) two years ago, with the complete resolution of symptoms following appropriate treatment. Family autoimmunity history was not reported.

**Figure 1:**
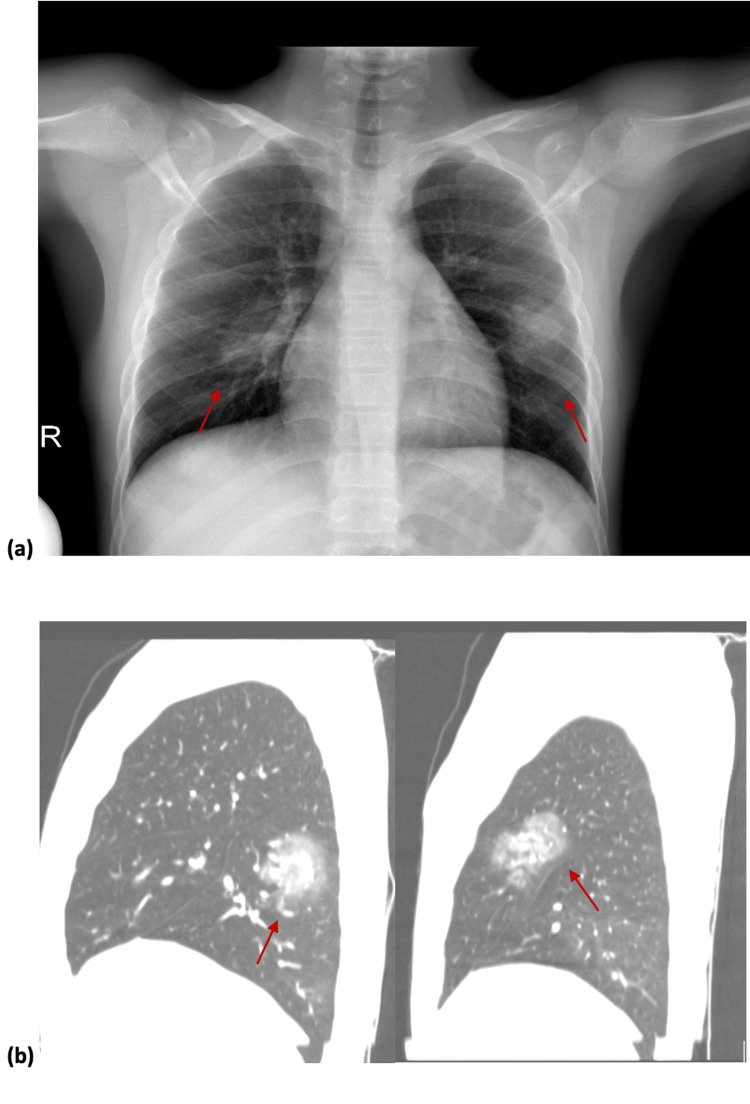
(a) Chest X-ray on admission showing patchy shadows in both lungs, (b) lung CT (sagittal plane) showing ground-glass patches on both lungs.

Detailed clinical examination revealed edema of the left lower limb and target-like hemorrhagic exanthem. Pulmonary examination revealed normal lung sounds. No clinical manifestations from the central or peripheral nervous system were detected. The ophthalmological and audiological examination was normal. Initial baseline laboratory investigations showed abnormal renal function tests (creatinine 1.3 mg/dl, urea 62mg/dl), increased ESR (90 mm/sec), and microscopic hematuria without proteinuria. SARS-CoV-2 antibody titers were 18900 AU/ml. Detailed humoral and cellular immunity tests, including angiitis-specific antibodies, showed MPO-ANCA seropositivity, and anti-GBM seronegativity, with normal peripheral blood smear immunophenotype. Abdominal ultrasound revealed bilateral kidney edema as well as pancreatic edema. Amylase and lipase levels were normal. Cranial CT was normal. Based on the aforementioned results, the patient underwent renal biopsy which revealed focal necrotizing and crescentic glomerulonephritis up to 50% of the glomeruli. Tubular atrophy and median fibrosis of the interstitium, without findings of necrotizing arteritis, were also described. On chest X-ray and CT (Figure 1), lung patchy shadows were detected. Lung function tests (spirometry, CO diffusion, plethysmography) were performed and restrictive lung disease with concomitant diffusion abnormalities were noted. As such, the diagnosis of MPO-ANCA angiitis was confirmed.

Based on the above findings, therapy including five intravenous methylprednisolone pulses (1gr/1.73 m2) was initiated. Simultaneously courses of cyclophosphamide were scheduled to be administered every two weeks, and eventually, the patient received six courses followed by rituximab maintenance therapy.

**Figure 2:**
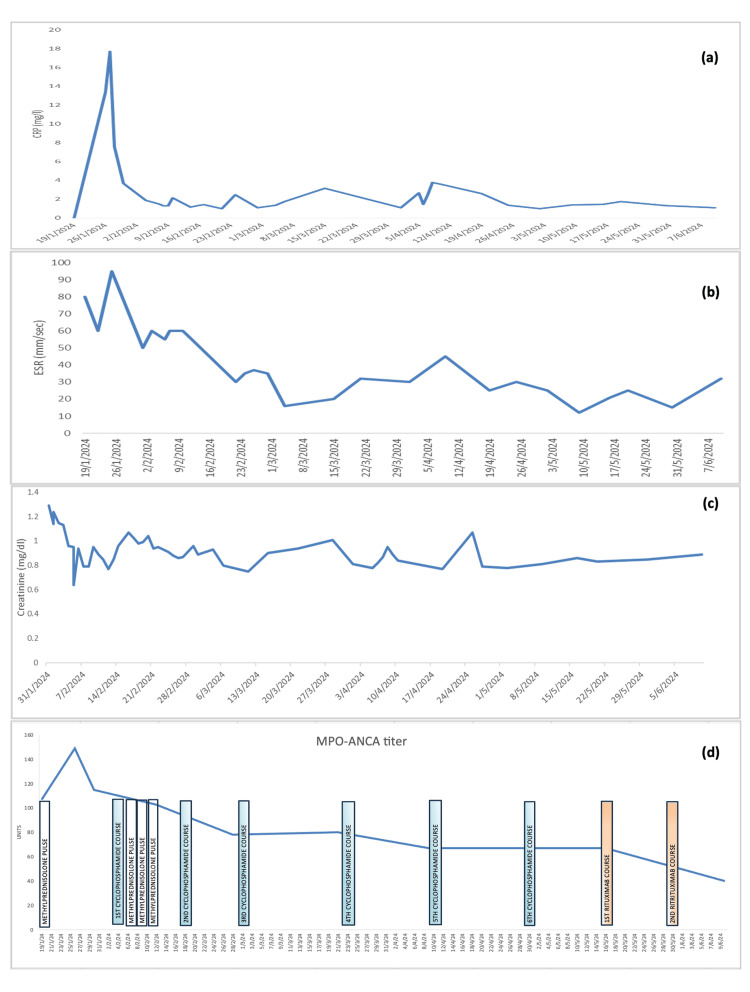
Fluctuation of CRP (a) ESR (b), creatinine (c), and MPO-ANCA levels (d) in accordance with the administrated therapeutic scheme.

On Day 5 of hospitalization, the patient complained of severe epigastric pain and developed massive hematochezia with anemia, necessitating a red blood cell transfusion. The abdominal ultrasound scan was repeated and showed an edematous intestinal wall which was attributed to intestinal small vessel inflammation. Two days following the hemorrhage recession, he presented with non-bilious vomiting. Laboratory tests revealed elevated amylase (750 U/l) and lipase levels (1300 IU/l) and discontinuation of feeding was suggested. The patient clinically improved after the second course of cyclophosphamide, with normalization of renal and pancreatic function tests. Post-discharge, pancreatic function tests normalized, but renal tests indicated acute kidney disease (creatinine: 0.9 mg/dl, urea: 60 mg/dl, GFR 73ml/min/1.73m2) (Figure 2, Table 1). Microscopic hematuria was persistent (urine red blood cells ranging between 5-80 cells/μl). The patient developed mild proteinuria (<500mg per day) which was treated with ramipril, an angiotensin-converting enzyme (ACE) inhibitor. The patchy shadows in the lungs disappeared after the first cyclophosphamide course. The patient was discharged on the 45th day of hospitalization with weekly follow-up tests arrangement. He continued with a corticosteroid tapering program.

**Table 1:** Values of creatinine, ESR, CRP, MPO-ANCA titers, amylase, and lipase on admission and after every therapy course received by the patient.

Variables	Admission	First cyclophosphamide course	Second cyclophosphamide course	Third cyclophosphamide course	Fourth cyclophosphamide course	Fifth cyclophosphamide course	Sixth cyclophosphamide course	First rituximab dose	Second rituximab dose	Reference ranges
Creatinine	1.29	1.24	0.95	0.91	0.90	0.78	0.79	0.86	0.85	0.2-1 mg/dl
ESR	80	95	60	30	20	45	30	21	15	<10 mm/sec
CRP	25	13.7	2.13	1.09	3.15	2.65	1.36	1.44	1	<1 mg/l
MPO-ANCA titer	107	149	115	78	80	67	67	67	40	<30 units
Amylase	90	85	1241	312	215	132	139	115	123	30-120 U/L
Lipase	57	1304	1437	176	95	51	37	48	40	<55 IU/L

## Discussion

To the best of our knowledge, this is the first reported case of a newly diagnosed MPO-ANCA-positive vasculitis following a previously confirmed MIS-C diagnosis and the second reported case associated with SARS-CoV-2 infection [6]. In the literature, there is only one report of a new onset anti-PR3 ANCA vasculitis after asymptomatic COVID-19 [7].

Our patient was initially diagnosed with MIS-C at the age of 12 years and presented with MPO-ANCA angiitis at the age of 14 years. Based on current evidence, there are two more pediatric cases of patients aged 12 and 17 years old respectively, with MPO-ANCA vasculitis following COVID-19 presenting two months later with diffuse alveolar hemorrhage and acute kidney injury possibly as a subsequent postinfectious development of anti-MPO vasculitis [6,8]. In a recent review where all forms of acute and late-onset vasculitis associated with COVID-19 in children were included, the time interval between infection and vasculitis development fluctuated from one week to five months [8]. In our case, not only was vasculitis developed even later than the aforementioned time intervals (at approximately two years post-MIS-C), but also other involved systems are rarely encountered in children with vasculitis, since skin, joints, pancreas, and gastrointestinal wall were additionally affected [9]. Gastrointestinal, kidney, and pulmonary manifestations are rare but life-threatening complications of MPO-ANCA vasculitis. In a 23-year-long, large-scale study involving patients with either MPO- or PR3-ANCA vasculitis, kidney, lung, and gastrointestinal involvement were reported in only 114, 72, and 5 patients, respectively, thus highlighting the rarity of these complications [9].

The patient was diagnosed with MIS-C two years before admission; additionally, when he presented with vasculitis symptoms, he had higher SARS-CoV-2 antibody titers compared to the first hospitalization (780 vs 18900 AU/ml). We hypothesize that our patient had a breakthrough SARS-CoV-2 infection between his two hospitalizations that could have triggered the onset of vasculitis symptoms. Since the initial reports of the condition in 2020, the hypothesis that links MIS-C to immunological dysregulation caused by SARS-CoV-2 infection has been substantiated [10]. The link between MIS-C and autoimmune diseases is even more supported, given that both are more common in children >5 years and especially adolescents [10].

The development of autoimmunity, following SARS-CoV-2 infection has been attributed to persistence of the virus, latent virus reactivation, or prolonged tissue damage due to chronic inflammation [11]. There are different theoretical explanations for the development of autoimmunity in patients with SARS-CoV-2 infection such as: (1) superantigen activity: the S protein of SARS-CoV-2 contains sequence and structure motifs similar to those of a bacterial superantigen and can bind directly to the T-cell receptor; (2) molecular mimicry and overt autoimmunity: accumulating evidence demonstrates that the virus has structural similarity to host-derived components. In addition, SARS-CoV-2 could induce hyperactivation of the immune system, leading to the synthesis of multiple autoantibodies in patients with severe SARS-CoV-2 infection. (3) Neutrophil extracellular traps (NETs): excessive neutrophil recruitment, activation, degranulation, and release of NETs can serve as a source of autoantigens resulting in the development of autoimmune conditions. (4) Type I interferon (IFN) response: SARS-CoV-2 induces the expression of numerous IFN-stimulated genes that exhibit immunopathogenic potential with overexpression of genes involved in inflammation [12-14].

Another possible mechanism that correlates SARS-CoV-2 with vasculitis development could be the viral tethering to the ACE-2 receptor on human cells. The ACE-2 receptor, widely located in various tissues including the lungs, cardiovascular system, gut, kidneys, central nervous system, and adipose tissue, plays a pivotal role in immunity and inflammation [15]. Chen et al. pinpointed the role of ACE-2 receptor and its polymorphisms in MIS-C development [16,17]. SARS-CoV-2 binds to the ACE-2 receptor and enters the endothelial cells, activating an inflammatory cascade with cytokines production and coagulation abnormalities [18,19]. Thrombotic microangiopathy, endothelial cell damage, fibrinoid necrosis, abnormalities in the coagulation/fibrinolytic system, and monocyte-derived macrophages and neutrophils are all outcomes of post-infectious inflammatory response that follows infection [20]. In this inflammatory milieu, ANCA is produced by recruited neutrophils and macrophages as new epitopes are formed [19-21]. Cytokines, active neutrophils, and macrophages contribute to the production of ANCA, and it is widely believed that AAV is caused by the induction of vasculitis by neutrophil extracellular traps [21].

IL-17 and IFN-gamma levels may be engaged in MIS-C inflammatory pathways due to the increase of CD4- and CD8- producing IL-17 in acute MIS-C patients and the decrease in convalescent MIS-C [22,23]. Th17 cells release IL-17A, which increases the production of proinflammatory cytokines such as IL-17, IL-22, and IL-26, resulting in enhanced neutrophil recruitment and cardiovascular complications [24]. Immune-mediated chronic inflammatory disorders such as psoriasis, ankylosing spondylitis, rheumatoid arthritis, and Crohn's disease are also related to IL-17 increase [25]. Even though cytokine levels were not investigated in our case, it is possible that post-MIS-C MPO-ANCA vasculitis could be attributed either to the initial paramount increase of IL-17 and IFN-gamma levels during MIS-C onset or to the persistently high cytokine levels at vasculitis diagnosis. Therefore, future studies should focus on the activated common immunological pathways and gene expression profiles between MIS-C and MPO-ANCA vasculitis patients.

The association between SARS-CoV-2 infection and future development of autoimmunity has been investigated and a recent study by Tesch et al. has found that the additional risk for any newly diagnosed autoimmune disease in patients with SARS-CoV-2 infection was 4.50 per 1000 person-years [26]. The authors have studied patients up to 15 months after infection and have also reported that SARS-CoV-2 infection was strongly associated with vascular autoimmune diseases. Two other studies from the United Kingdom and the United States have also reported an association between SARS-CoV-2 infection and the development of autoimmunity [27,28] but these studies have included patients older than 18 years of age. In the UK retrospective study, the patients should have been registered in the database for at least 12 months for sufficient data collection. In the US study, the patients' follow-up period was set from the start point of 30 days after infection up to six months. The absolute incidence rates (IR) of any autoimmune disease were higher in female and older patients, and among those without preexisting autoimmune disease [26].

Intravenous corticosteroids and cyclophosphamide are first-line intravenous therapeutic options in patients with SARS-CoV-2-associated AAV [29,30]. In the literature, it is apparent that most patients have received corticosteroids (40%), while rituximab (14.2%) and cyclophosphamide (11.4%) were the most frequently used immunosuppressive drugs [8]. Antithrombotic treatment is only suggested for AAV patients with venous thrombotic events. 

Although in the other two case reports, there was a complete resolution of the symptoms after treatment, our patient continues to have persistent kidney injury and has also developed restrictive lung disease. There are not many studies on the outcome of COVID-19-associated vasculitis and it is reported that remission was achieved in 23 of 28 adult patients, but five patients died (four of central nervous system vasculitis and one of ANCA-associated vasculitis) [8].

## Conclusions

To our knowledge, this is the first reported case of MPO-ANCA vasculitis in a pediatric patient with a previous medical history of MIS-C and a second SARS-CoV-2 breakthrough infection confirmed by increased SARS-CoV-2 antibody titers. Although acute complications of SARS-CoV-2 are well documented, long-term complications are still not well elucidated, especially the risk of the development of autoimmunity. More research is also required to clarify further the link between severe SARS‐CoV‐2 infection and autoimmune disorders trigger potential.
